# *Docynia delavayi* polyphenols enhance short-chain fatty acid synthesis *via* the chlorogenic acid–caffeic acid–protocatechuic acid pathway: insights from *in vitro* digestion–fermentation^☆^

**DOI:** 10.1016/j.fochx.2025.103416

**Published:** 2025-12-16

**Authors:** Tingting Zhang, Mingxia Xing, Hui Zhang, Xin Song, Zibo Song, Chunmei Yuan, Jun Zhang, Zhou Zhang, Fan Xie, Lianzhong Ai

**Affiliations:** a Shanghai Engineering Research Center of Food Microbiology, School of Health Science and Engineering, University of Shanghai for Science and Technology, Shanghai, China; b Department of Food Science & Technology, School of Agriculture and Biology, Shanghai Jiao Tong University, 800 Dongchuan Road, Shanghai, China; c Shidong Hospital, Yangpu District, Shanghai, China; d Yunnan Provincial Key Laboratory of Applied Technology for Special Forest Fruits, Yunnan Maoduoli Group Food Co., Ltd., Yuxi, China

**Keywords:** Docynia delavayi, Gut microbiota, Short-chain fatty acids, *In vitro* digestion, Colonic fermentation, Caffeic acid, Protocatechuic acid

## Abstract

*Docynia delavayi* fruit polyphenols (DDP) demonstrate potential for enhancing short-chain fatty acid (SCFA) synthesis; however, underlying mechanisms remain poorly understood. This study utilized an *in vitro* digestion–fermentation model combined with multi-omics analyses to explore these mechanisms. The *in vitro* model revealed notable alterations in both 1,1′-diphenyl-2-picrylhydrazyl and 2,2′-azino-bis(3-ethylbenzothiazoline-6-sulfonic acid) radical-scavenging capacities, as well as in total phenolic and flavonoid content, accompanied by increased production of acetic, propionic, and butyric acids. Metagenomic indicated that DDP stimulated *Bifidobacterium adolescentis*, *Bifidobacterium pseudocatenulatum*, *Bifidobacterium longum*, and *Bifidobacterium bifidum* growth. Metabolomics demonstrated enrichment of SCFA-associated metabolic pathways, including propanoate and butyrate metabolism, and identified caffeic acid and protocatechuic acid as primary bioactive metabolites produced from DDP. Multi-omics analysis suggested that DDP modulated gut microbiota by enriching the chlorogenic acid–caffeic acid–protocatechuic acid metabolic pathway (*r* > 0.95, *p* < 0.01), ultimately boosting SCFA biosynthesis. This study offers new insights into the mechanisms by which polyphenols regulate health.

## Highlights

• *D. delavayi* polyphenols (DDPs) boost bioactivity during *in vitro* digestion and fermentation.
• DDPs significantly enhanced short-chain fatty acid (SCFA) production.
• DDPs improved gut microbiota and their metabolic profile.
• DDPs promote SCFA production *via* the chlorogenic acid-caffeic acid-protocatechuic acid metabolic pathway.

## Graphical abstract

**Figure:**
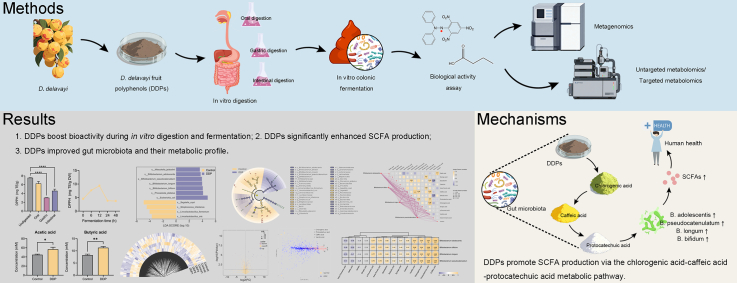

## 1 Introduction

The gut microbiota comprises the diverse microbial community residing in the digestive tract (Lynch & Pedersen, 2016). It plays essential roles in metabolic interactions, immune function, and physiological regulation. Through these mechanisms, the gut microbiota provides a broad range of health benefits. In terms of metabolic regulation, the gut microbiota contributes 5–10 % of daily energy requirements, synthesizes B vitamins, neurotransmitters, and other beneficial compounds, and assists in exogenous toxin degradation (Lynch & Pedersen, 2016). Regarding immune regulation, the microbiota supports the differentiation and maturation of immune cells, influences enzyme systems, such as cyclooxygenase-2 (COX-2) and inducible nitric oxide synthase (iNOS), modulates signaling pathways (including nuclear factor [NF]-κB and mitogen-activated protein kinase [MAPK]), and helps maintain gut barrier integrity (Sanz et al., 2025). Physiologically, the gut microbiota impacts cell proliferation, glucose and lipid absorption, intestinal endocrine function, and neural signaling (Lynch & Pedersen, 2016; Sanz et al., 2025).

Polyphenols are naturally occurring compounds in plants that constitute the most abundant class of dietary antioxidants. Nearly 10,000 polyphenolic compounds have been identified, predominantly in plant-based foods such as fruits, vegetables, and grains (Wan et al., 2021). However, 90–95 % of these dietary polyphenols are not absorbed in the upper gastrointestinal tract and instead reach the colon, where they are extensively metabolized by the gut microbiota (Wan et al., 2021). The microbial metabolism of dietary polyphenols is a pivotal element in the diet–microbe–host co-metabolic system, characterized by a bidirectional interaction. In this process, the gut microbiota converts polyphenols into low-molecular-weight metabolites through mechanisms such as deglycosylation, dehydroxylation, and ring cleavage. These transformations enhance the bioavailability and activity of polyphenols, ultimately influencing host health. For example, millet-derived polyphenols are metabolized into quinic acid, which alleviates oxidative damage in high-fat diet-induced mouse models (Li et al., 2024). Similarly, *Enterocloster* species convert urolithins into urolithin A *via* molybdenum cofactor-dependent enzymes by cleaving the 9-hydroxyl group, thereby enhancing cholesterol metabolism and attenuating inflammation (Pidgeon et al., 2025). Additionally, polyphenols can exert prebiotic effects by promoting beneficial microbes and inhibiting pathogenic bacteria. For instance, *Prunus persica* polyphenols restore microbial balance and improve gastrointestinal motility in constipation models (Liang et al., 2024), while polyphenols from Liubao insect tea help restore gut barrier integrity and reduce intestinal inflammation by modulating the relative abundances of *Akkermansia* and *Clostridia* (Feng et al., 2025). These interactions support intestinal health by stimulating the production of bioactive metabolites, stabilizing microbial structure, and preserving gut homeostasis.

*Docynia delavayi* (Franch.) Schneid is a perennial deciduous plant belonging to the Rosaceae family, widely distributed across the high-altitude regions of southwestern China. Its fruits are widely utilized in traditional medicine and food processing (Tian et al., 2024). Compared with common fruits like apples and pears, *D. delavayi* fruits contain distinct phytochemical profiles, including high levels of polyphenols (Tian et al., 2023). We previously employed ultrasound-assisted extraction to obtain high-purity *D. delavayi* fruit polyphenols (DDP) and optimized the process using response surface methodology. The primary constituents of DDP were also identified, and their therapeutic potential was evaluated in a mouse model of colitis (Zhang et al., 2025). DDP are particularly rich in procyanidin B2, vitexin, and myricitrin. Supplementation with DDP significantly increased short-chain fatty acid (SCFA) levels—notably acetate, propionate, and butyrate—in a mouse colitis model, and this increase was associated with improved gut barrier integrity and reduced inflammation. However, the precise mechanisms driving these effects remain unclear.

Given that SCFAs are predominantly generated through microbial metabolism in the colon (Mukhopadhya & Louis, 2025), we hypothesized that DDP promote SCFA synthesis by modulating gut microbiota composition and function. However, the specific effects of DDP on the gut microbiota, as well as the identities of its microbial metabolites, remain unclear. Additionally, as the bioactivity of DDP is largely mediated by the microbial metabolites (Di Lorenzo et al., 2021)—such as the conversion of procyanidins into phenolic acids—it is essential to identify the key bioactive compounds. Addressing these knowledge gaps is necessary to fully harness DDP in functional food development and to integrate them into personalized nutrition strategies.

*In vitro* digestion and colonic fermentation models are widely used experimental systems for investigating dietary nutrients (Ismail et al., 2021; Li et al., 2024; Wang et al., 2025). These models offer several advantages, including cost-effectiveness, high controllability, minimal ethical concerns, and flexibility, making them ideal for investigating the dynamics of polyphenol release and microbial metabolism within the gastrointestinal environment. Additionally, these models enable the real-time monitoring of polyphenol-induced changes in the gut microbiota, providing insights into the molecular mechanisms underlying polyphenol–microbiota interactions. Accordingly, this study employed an integrated approach that combines *in vitro* digestion–colonic fermentation models with multi-omics technologies to systematically investigate the digestive release kinetics of DDP, evaluate their regulatory effects on the structure and function of gut microbiota, and assess their impact on the microbial metabolome. Furthermore, the study sought to elucidate the mechanisms by which DDP contribute to intestinal health. The findings are expected to provide a theoretical foundation for developing polyphenol-based functional foods and microbiota-targeted therapeutics, while promoting the value-added utilization of endemic plant resources from Southwest China.

## 2 Materials and methods

### 2.1 Plant materials and polyphenol extraction procedures

Fresh, mature, and, disease-free *D. delavayi* fruits were sourced from Pu’er City, Yunnan Province, China. After washing, the fruits were homogenized in a high-speed blender and immediately lyophilized. The lyophilized material was ground into a fine powder and passed through a 100-mesh sieve. For extraction, 150 g of the freeze-dried powder was combined with 43 % ethanol at a liquid-to-solid ratio of 21 mL/g. Ultrasound-assisted extraction was performed at 40 kHz and 460 W for 41 min. The resulting mixture was centrifuged at 7000 ×*g* and 4 °C for 5 min. The supernatant was collected, filtered, and concentrated under vacuum at 55 °C. Subsequently, the concentrate was loaded onto an AR31 macroporous resin chromatography column (ϕ 10 cm × 100 cm; bed volume: 10 L). Four bed volumes of deionized water were used to remove impurities, followed by elution with three bed volumes of 95 % ethanol to obtain the target polyphenols. The ethanol eluate was concentrated and lyophilized to obtain the DDP powder.

### 2.2 *In vitro* simulated gastrointestinal digestion

The *in vitro* digestion process followed the standardized static INFOGEST 2.0 protocol developed by Brodkorb et al. (2019), encompassing sequential oral, gastric, and intestinal digestion stages. The simulated salivary fluid (SSF), gastric fluid (SGF), and intestinal fluid (SIF) were prepared and used at concentrations consistent with published protocols (Brodkorb et al., 2019). The SSF comprised 15.1 mM KCl, 3.7 mM KH_2_PO_4_, 13.6 mM NaHCO_3_, 0.15 mM MgCl_2_·6H_2_O, 0.06 mM (NH_4_)_2_CO_3_, 1.5 mM CaCl_2_·2H_2_O, and 75 U/mL salivary α-amylase, adjusted to pH 7 with HCl. The SGF included 6.9 mM KCl, 0.9 mM KH_2_PO_4_, 25 mM NaHCO_3_, 47.2 mM NaCl, 0.10 mM MgCl_2_·6H_2_O, 0.5 mM (NH_4_)_2_CO_3_, 0.15 mM CaCl_2_·2H_2_O, and 2000 U/mL pepsin, adjusted to pH 3. The SIF was composed of 6.8 mM KCl, 0.8 mM KH_2_PO_4_, 85 mM NaHCO_3_, 38.4 mM NaCl, 0.33 mM MgCl_2_·6H_2_O, 0.6 mM CaCl_2_·2H_2_O, 100 U/mL trypsin, and 1.75 mM bile salts, adjusted to pH 7.

Critical components such as CaCl_2_, enzymes, and bile salts were added immediately before use to ensure effectiveness. Electrolyte solutions were preheated to 37 °C to mimic physiological temperature.

During the oral phase, DDP were dissolved in deionized water (4 mg/mL) and mixed with an equal volume of SSF, followed by incubation at 37 °C for 2 min. For the gastric phase, the oral digest was combined with an equal volume of SGF and incubated at 37 °C for 2 h. In the intestinal phase, the gastric digest was combined with an equal volume of SIF and incubated at 37 °C for 2 h. Samples from each phase were collected for subsequent analysis. Following each stage, samples were centrifuged to isolate the precipitate, which was then lyophilized for further use.

### 2.3 *In vitro* colonic fermentation

*In vitro* colonic fermentation followed a slightly modified version of the protocol by Pérez et al. (2021) and received approval from the Ethics Review Committee of Shandong First Medical University and Shandong Academy of Medical Sciences (approval number R2024070520333, June 26, 2024). All procedures adhered to institutional and legal guidelines. Fecal samples were collected from four healthy adult volunteers (two males and two females, aged 20–35 years), none of whom had taken antibiotics or had gastrointestinal disease or surgery within the previous three months. Informed consent was obtained from all participants, and their privacy was protected throughout the study.

The stool samples were pooled in equal proportions and diluted with sterile saline containing 0.50 g/L cysteine hydrochloride to a 10 % (*w*/*v*) concentration, followed by filtration through eight layers of sterile gauze to yield the fecal filtrate. A modified yeast casitone fatty acids (YCFA) broth was prepared as detailed in Table S1. For the DDP group, the modified YCFA broth was supplemented with 10 % fecal filtrate and 100 mg of lyophilized residue from the simulated gastrointestinal digestion. The control group was prepared similarly, excluding the digestion residues. Both groups were incubated under anaerobic conditions (5 % CO₂, 10 % H₂, 85 % N₂) for 48 h. Samples were collected at 0, 6, 12, 24, and 48 h for analysis.

### 2.4 Determination of total phenolic content (TPC) and total flavonoid content (TFC)

The TPC and TFC of DDP were measured during the simulated gastrointestinal digestion phases (undigested, oral, gastric, and intestinal) and throughout colonic fermentation (0, 6, 12, 24, and 48 h), following the protocol of Ismail et al. (2021). For TPC measurement, a 0.20 mg/mL gallic acid standard was serially diluted to 8, 12, 16, 20, 24, and 30 μg/mL. A 100-μL aliquot of each dilution was mixed with 100 μL of 0.1 mol/L Folin–Ciocalteu reagent and incubated at 25 °C for 3 min. After adding 10 μL of 10 % (*w*/*v*) sodium carbonate, the solution was agitated every 10 min for 1 h. The absorbance at 720 nm was measured using an SPZ-009 microplate reader (Thermo Fisher Scientific, Waltham, MA, USA), and a standard curve was constructed. Sample absorbance was measured using the same protocol, with 50 % ethanol as the blank control. TPC was expressed as gallic acid equivalents (GAE, mg GAE/g dry weight [DW]).

For TFC determination, a rutin standard was diluted to 1.25, 2.5, 5, 10, 15, 20, 22.5, and 25 μg/mL. A 20-μL aliquot of each standard dilution was mixed with 40 μL of 0.1 mol/L aluminum chloride, 60 μL of 1 mol/L potassium acetate, and 80 μL of 70 % methanol. After thorough mixing, the solution was incubated at 25 °C for 30 min, and the absorbance at 420 nm was recorded. A standard curve was plotted and used to calculate sample TFC values, expressed as rutin equivalents (RE, mg RE/g DW), with 70 % methanol as the blank control.

### 2.5 Determination of antioxidant activity

The antioxidant capacity of DDP was evaluated by measuring the 1,1′-diphenyl-2-picrylhydrazyl (DPPH) radical scavenging activity and 2,2′-azino-bis(3-ethylbenzothiazoline-6-sulfonic acid) (ABTS) antioxidant capacity across the simulated digestion stages (undigested, oral, gastric, and intestinal phases), as well as during colonic fermentation (0, 6, 12, 24, and 48 h). Specialized reagent kits provided by the Nanjing Jiancheng Bioengineering Institute (Jiangsu, China) were used for all assays.

### 2.6 Determination of SCFA content

A 500 μL aliquot of the fermentation supernatant was collected after 48 h of *in vitro* fermentation. The sample was acidified with 10 % sulfuric acid, vortexed, and mixed with anhydrous ether. After centrifuging at 12,000 ×*g* for 15 min at 4 °C, the supernatant was combined with 0.25 g anhydrous sodium sulfate, incubated at 25 °C for 30 min, and centrifuged again. The final supernatants were filtered through a 0.22-μm membrane and stored for analysis. The concentrations of SCFAs were determined using gas chromatography (GCMS-QP2010 Ultra System, Shimadzu Corporation, Kyoto, Japan), with chromatographic separation performed on an Rtx-Wax capillary column. The temperature program began at 100 °C, increased to 140 °C at 7.5 °C/min, then to 200 °C at 60 °C/min, and was held for 3 min. Mass spectrometry (MS) was performed in full-scan mode (*m/z* 33–110), with external standards used to quantify acetic, propionic, butyric, isobutyric, valeric, and isovaleric acids.

### 2.7 Metagenomics analysis

The effect of DDP on the gut microbiota was assessed using metagenomic as described by Qu et al. (2025). After 48 h of *in vitro* fermentation, samples were collected for DNA extraction and quality assessment. DNA meeting QC thresholds was fragmented to 350 bp, end-repaired, A-tailed, and ligated to Illumina sequencing adapters. PCR amplification enriched the target fragments, which were then purified with an AMPure XP system. Library quality was assessed using an Agilent 2100 Bioanalyzer (Agilent Technologies, Böblingen, Germany) and quantified by real-time PCR. High-throughput sequencing was conducted on the NovaSeq X Plus platform. Sequencing reads were filtered using Fastp (v0.20.0) and assembled using Megahit (v1.1.2), followed by analysis of gut microbiota composition and intergroup differences. The metagenomic data have been deposited in the Genome Sequence Archive in BIG Data Center (https://bigd.big.ac.cn/), Beijing Institute of Genomics (BIG), Chinese Academy of Sciences, under the accession number: CRA034258.

### 2.8 Untargeted metabolomics analysis

An untargeted metabolomics approach, based on Zuo et al. (2025), was used to evaluate the impact of DDP on the gut microbial metabolic profiles. Fermented supernatants collected after 48 h of *in vitro* fermentation were mixed with a pre-cooled methanol/acetonitrile/water solution (2:2:1, *v*/v), ultrasonicated at 4 °C for 30 min, incubated at −20 °C for 10 min, and centrifuged at 14,000 ×*g* for 20 min at 4 °C. The supernatant was lyophilized, reconstituted in 100 μL of a 1:1 (*v*/v) acetonitrile/water mixture, and centrifugation at 14,000 ×*g* for 15 min at 4 °C. The final supernatants were collected for further analysis.

Metabolites were analyzed using an Agilent 1290 Infinity LC UHPLC system (Agilent Technologies, Inc., Santa Clara, USA), equipped with an ACQUITY UPLC BEH Amide column (1.7 μm, 100 × 2.1 mm; Waters, Milford, MA, USA) at 25 °C, with a flow rate of 0.5 mL/min, and a 2 μL injection volume. Mobile phases comprised 25 mM ammonium hydroxide and 25 mM ammonium acetate in water (A) and acetonitrile (B). The elution gradient was: 0–0.5 min, 95 % B; 0.5–7 min, 95–65 % B; 7–8 min, 65–40 % B; 8–9 min, hold at 40 % B; 9–9.1 min, 40–95 % B; 9.1–12 min, hold at 95 % B. MS was performed on an AB Triple TOF 6600 (AB Sciex Pte. Ltd., USA), with ion source gases 1 and 2 at 60 psi, curtain gas at 30 psi, source temperature at 600 °C, and spray voltage of ±5500 V. The time-of-flight mass spectrometer operated with an *m/z* scan range of 60–1000 Da, a declustering potential of ±60 V, and a collision energy of 35 ± 15 eV.

### 2.9 Targeted metabolomics analysis of phenolic acid compounds

Targeted metabolomic analysis of phenolic acid compounds was performed using the method established by Zuo et al. (2025). A comprehensive list of detected phenolic acid metabolites is provided in Table S2. Briefly, 100 μL of fermented supernatants collected after 48 h were mixed with 400 μL of methanol containing 1 % ascorbic acid, ultrasonicated at 4 °C for 30 min, and centrifuged at 14,000 ×*g* for 10 min at 4 °C. The supernatant was filtered before analysis. Separation was achieved on a BEH C18 column (1.7 μm, 100 × 2.1 mm; Waters) using a mobile phase of 0.1 % formic acid in water (A) and methanol (B), at 35 °C, flow rate of 0.3 mL/min, and injection volume of 1 μL. The gradient was: 0.0–1.0 min, 8 % B; 1.0–3.0 min, 8–12 % B; 3.0–5.0 min, hold at 12 % B; 5.0–8.5 min, 12–100 % B; 8.5–8.51 min, 100–8 % B; and 8.51–11.0 min, hold at 8 % B.

MS was performed on a QTRAP® 6500+ triple quadrupole mass spectrometer (AB SCIEX LLC, Framingham, USA) in positive and negative ion modes, with ion source gases at 55 psi, curtain gas at 35 psi, spray voltage at +5500 V (positive mode) or − 4500 V (negative mode), and source temperature at 300 °C.

### 2.10 Multi-omics analysis

Multi-omics analysis was conducted as described by Qu et al. (2025). SCFA data, gut microbiota composition, and phenolic acid levels were selected for multi-omics analysis. For correlation clustering and Mantel tests, datasets were transformed into matrix format, and Pearson correlation coefficients were calculated using the ggcorrplot package (v0.9.8.1) in R (v4.2.0). Canonical correspondence analysis (CCA) and redundancy analysis (RDA) were performed using the vegan package (v2.5.6) in R (v4.2.0).

### 2.11 Statistical analysis

Data are presented as means ± standard deviation (SD). Statistical significance was evaluated using one-way analysis of variance (ANOVA), followed by Tukey's *post hoc* test with GraphPad Prism software (v9.5.1). For untargeted metabolomics, heatmaps were generated from z-score-normalized data. Statistical significance was set at *p* < 0.05.

## 3 Results and discussion

### 3.1 Changes in the TPC and TFC of DDP during *in vitro* digestion and colonic fermentation

**Fig. 1:**
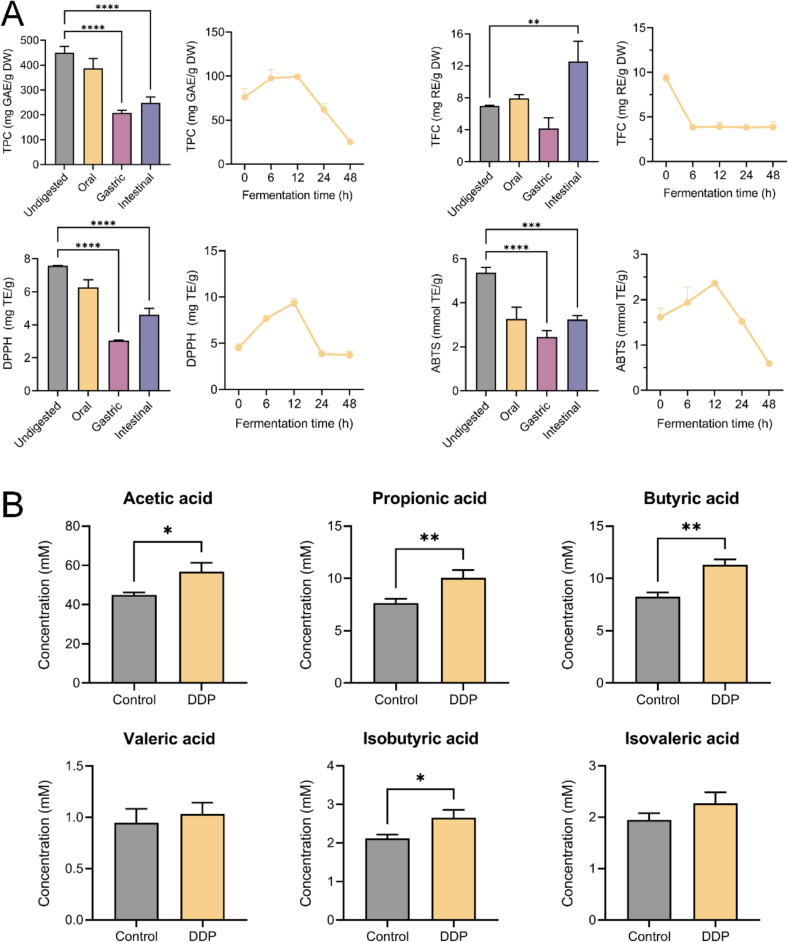
DDP boost bioactivity and enhance SCFA synthesis during *in vitro* digestion and fermentation. (A) Changes in total phenolic content (TPC), total flavonoid content (TFC), DPPH radical scavenging activity, and ABTS radical scavenging activity during *in vitro* digestion and colonic fermentation; (B) Levels of SCFAs after 48 h fermentation. DDP, polyphenols from *Docynia delavayi* (Franch.) Schneid fruit; SCFAs, short-chain fatty acids. *p < 0.05; **p < 0.01; *n =* 3.

Fig. 1A illustrates the dynamic changes in the TPC and TFC of DDP during *in vitro* digestion and fermentation. Relative to the undigested sample, the TPC significantly decreased following simulated gastrointestinal digestion. A detailed analysis of each digestion stage revealed that TPC levels remained relatively stable during the oral phase (449.73 mg GAE/g DW). This stability is attributable to the short exposure time to salivary amylase, which exerts minimal catalytic activity on phenolic constituents (Ismail et al., 2021). The most pronounced decrease in TPC occurred during the gastric and intestinal phases (207.15 and 247.69 mg GAE/g DW, respectively; *p* < 0.001), consistent with prior findings regarding other polyphenols, such as proanthocyanidins and quinoa polyphenols, during *in vitro* digestion (Li et al., 2024; Wang et al., 2025). These declines may be attributed to two primary factors: pH fluctuations during digestion that cause polyphenol depolymerization under acidic conditions, resulting in smaller molecules and reduced TPC levels (Ismail et al., 2021), and the formation of protein–polyphenol complexes via digestive proteases, which reduce the availability of free polyphenols and interfere with TPC quantification (Chen et al., 2024).

During colonic fermentation, microbial metabolism became the dominant factor influencing TPC. The TPC initially increased, peaking at 12 h (99.30 mg GAE/g DW), before gradually declining throughout the fermentation period. This pattern underscores the crucial role of gut microorganisms in DDP metabolism. The gut microbiota secretes enzymes, such as cellulase and pectinase, which facilitate the breakdown of food matrices and the release of matrix-bound polyphenols (Zhang et al., 2019). Additionally, microbes hydrolyze complex polyphenolic polymers into simpler phenolic compounds (Zhang et al., 2019), contributing to the observed rise in TPC during the first 12 h. In contrast, the TPC decreased from 12 to 48 h, likely due to further microbial degradation of phenolic compounds. Indeed, TPC can be markedly depleted during the later stages of *in vitro* fermentation and may be fully metabolized within 48 h (Chait et al., 2020). The physiological effects of dietary polyphenols are largely mediated by microbial metabolites (Di Lorenzo et al., 2021). After microbial metabolism in the gut, polyphenols are converted into bioactive compounds such as phenolic acids, which improve bioavailability and functional activities, including free radical scavenging. This transformation is a key mechanism by which dietary polyphenols modulate gut microbiota composition (Di Lorenzo et al., 2021). The marked decrease in TPC during the later fermentation stages further confirms the efficient microbial metabolism of DDP and its associated health benefits.

TFC content increased during the oral phase, decreased in the gastric phase, and increased again during the intestinal phase (Fig. 1A). Although the oral-phase increase was not statistically significant, factors such as saliva-induced dissolution and release of flavonoids from the food matrix, enzymatic hydrolysis of glycosylated flavonoids, and structural stability of certain molecules during salivary digestion may have contributed to this trend (Laib et al., 2023). Similar to TPC, TFC decreased in the gastric phase due to acidic degradation and fragmentation of flavonoids into smaller components. During the intestinal phase, hydrolysis converts flavonoid glycosides into aglycones, resulting in a significant increase in TFC (Laib et al., 2023). During colonic fermentation, TFC decreased sharply within the first 6 h (*p* < 0.0001) and then stabilized. This decrease is likely attributable to the structural instability of flavonoids, as colonic microbes catalyze biotransformation reactions, including dehydroxylation, ring cleavage, and methylation (Zhou et al., 2024), which convert flavonoid aglycones into smaller bioactive metabolites (such as phenolic acids) and disrupt the flavonoid backbone.

### 3.2 Changes in the antioxidant activity of DDP during *in vitro* digestion and colonic fermentation

Evaluation of DDP's antioxidant activity revealed similar trends in DPPH radical scavenging activity and ABTS antioxidant capacity throughout the digestion and fermentation phases (Fig. 1A), exhibiting an initial decline during the oral and gastric phases, followed by recovery during the intestinal phase. By the end of digestion, DPPH radical scavenging activity and ABTS radical scavenging capacity reached 4.62 mg TE/g and 3.24 mmol TE/g, respectively. These patterns closely paralleled changes in TPC, as expected, since digestive proteases promote the formation of protein–polyphenol complexes, decreasing the availability of free polyphenols and reducing antioxidant capacity (Chen et al., 2024). Moreover, the acidic environment in the oral and gastric phases accelerates the degradation of phenolic compounds, further diminishing antioxidant effectiveness (Chen et al., 2024; Ismail et al., 2021). Collectively, these shifts reflect the degradation and biochemical transformation of DDP and confirm a notable reduction in antioxidant potential compared with the undigested state.

During colonic fermentation, antioxidant activity initially increased, then declined. Among the five time points evaluated, the peak occurred at 12 h, with DPPH and ABTS values reaching 9.34 mg TE/g and 2.37 mmol TE/g, respectively. This increase corresponded with the peak in TPC observed during fermentation. As TPC decreased, antioxidant activity also declined, indicating progressive depletion of bioactive compounds over time (Zhang et al., 2019).

### 3.3 DDP promoted SCFA synthesis

We have previously reported that DDP significantly enhance SCFA synthesis in the colitic gut of mice (Zhang et al., 2025). To further substantiate this effect, herein we quantified SCFA concentrations in the Control and DDP groups following *in vitro* fermentation (Fig. 1B). Of the six SCFAs detected, DDP significantly increased the production of acetic acid (44.89 *vs.* 56.80 mM, *p* < 0.05), propionic acid (7.63 *vs.* 10.06 mM, *p* < 0.05), butyric acid (8.26 *vs.* 11.29 mM, *p* < 0.05), and isobutyric acid (2.12 *vs.* 2.65 mM, *p* < 0.05). Although DDP also upregulated valeric acid synthesis, the change was not significant in this *in vitro* model, unlike in animal models (Zhang et al., 2025).

SCFAs are bioactive small molecules with notable health benefits. In the colon, they are primary energy sources for intestinal epithelial cells. Under inflammatory conditions, SCFAs support immune cell differentiation, help restore intestinal barrier integrity, and regulate metabolic processes (Mukhopadhya & Louis, 2025). Given that SCFA production is primarily mediated by the gut microbiota (Mukhopadhya & Louis, 2025), the composition and metabolic profile of these microbes are essential determinants of SCFA synthesis. Recent studies suggest that dietary factors can act as substrates or regulatory signals, stimulating the gut microbiota and altering specific species and metabolic functions (Mukhopadhya & Louis, 2025). Given DDP's observed effects on SCFA levels, it likely enhances SCFA synthesis by influencing the structure and metabolic activity of the gut microbiota. This underscores the importance of further investigation into the effects of DDP on gut microbiota.

### 3.4 DDP altered gut microbiota composition and enriched SCFA-producing species

**Fig. 2:**
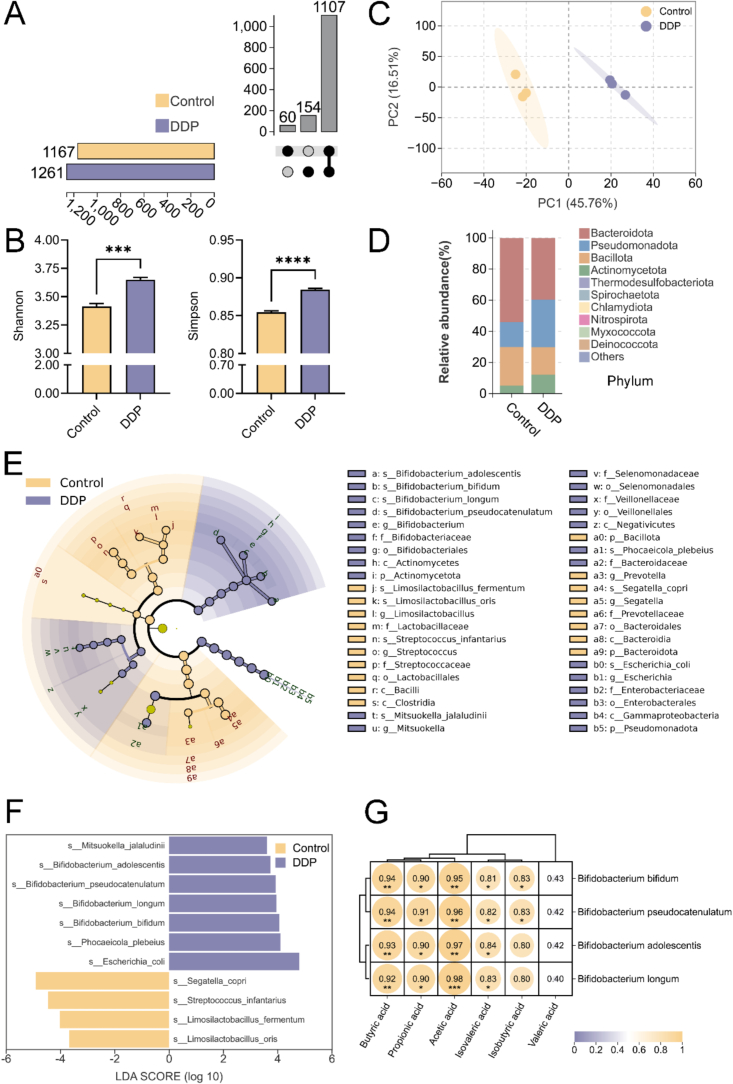
DDP improve gut microbiota composition. (A) UpSet plot of the gut microbiota at the species level. (B) Shannon and Simpson indices at the species level. (C) PCA at the species level; (D) Top 10 species by relative abundance at the phylum level. (E) Taxonomic cladogram in the LEfSe analysis (LDA score > 3.5). (F) LEfSe analysis of differential species between the Control and DDP groups (LDA score > 3.5). (G) Correlation analysis of SCFAs and the enriched *Bifidobacterium* species in the DDP group. DDP, polyphenols from *Docynia delavayi* (Franch.) Schneid fruit; SCFAs, short-chain fatty acids; PCA, principal component analysis. ***p < 0.001; ****p < 0.0001; *n =* 3.

Metagenomic analysis revealed a comprehensive overview of the gut microbiota following DDP intervention, identifying 18 phyla, 36 classes, 80 orders, 159 families, 377 genera, and 1321 species. At the species level, 1167 were identified in the Control group and 1261 in the DDP group, with 1107 species shared between the two groups (Fig. 2A). The α-diversity of the gut microbiota, assessed by the Shannon and Simpson indices, significantly increased after DDP intervention (Fig. 2B, *p* < 0.05), indicating enhanced microbial diversity and richness. Principal component analysis (PCA) further showed that DDP significantly increased microbial scores along the first principal component (PC1), while scores for PC2 remained unchanged (Fig. 2C). At the phylum level (Fig. 2D), Bacteroidetes, Bacillota, and Pseudomonadota predominated in both groups; however, the Bacillota/Bacteroidetes ratio did not differ significantly between them.

To assess the beneficial effects of DDP on the gut microbiota, linear discriminant analysis (LDA) effect size (LEfSe) analysis was performed using an LDA score threshold of >3.5 to identify differentially abundant species between the Control and DDP groups. DDP significantly modulated the gut microbial composition (Fig. 2E, F). Notably, the relative abundances of *Streptococcus infantarius* and *Segatella copri* were markedly reduced in the DDP group. Both species are opportunistic pathogens associated with infections, systemic inflammation, and chronic metabolic disorders, suggesting that DDP contribute to improved gut microbial homeostasis. Conversely, the relative abundances of several beneficial species—including *Mitsuokella jalaludinii*, *Bifidobacterium adolescentis*, *Bifidobacterium pseudocatenulatum*, *Bifidobacterium longum*, *Bifidobacterium bifidum*, and *Phocaeicola plebeius*—were significantly increased. These microbes are integral to intestinal health. For example, *M. jalaludinii*, a common gut resident, possesses high phytase activity, facilitating the degradation of phytic acid to inositol and phosphate, and producing intermediates that support gut function (De Vos et al., 2024). Similarly, *P. plebeius* demonstrates notable adaptability and a unique capacity to metabolize algal polysaccharides, positioning it as a key contributor to microbial interactions and host gut regulation (Kitahara et al., 2005). The increased abundance of various *Bifidobacterium* species—well-established probiotics—further highlights the role of DDP in promoting gut health. These bacteria produce bioactive metabolites, modulate immune responses, reinforce intestinal barriers, and help mitigate inflammation (Gavzy et al., 2023). Dietary polyphenols selectively suppress the growth of pathogenic bacteria while stimulating the growth of beneficial strains (Wan et al., 2021), a trend confirmed by the current findings.

The gut microbiota is the primary source of SCFAs (Mukhopadhya & Louis, 2025). Comparisons of microbiota composition between the DDP and Control groups revealed that *Bifidobacterium* species—including *B. adolescentis*, *B. pseudocatenulatum*, *B. longum*, and *B. bifidum*—were substantial contributors to SCFA production in the DDP group. Acetic acid, a primary metabolic product of *Bifidobacterium,* is among the most abundant metabolites released into the systemic circulation (Vieira et al., 2016). Several acetic acid-producing *Bifidobacterium* strains can metabolize diverse carbohydrates and possess efficient carbohydrate transport systems, enabling substantial acetate production in the distal colon (Gavzy et al., 2023). Regarding propionate and butyrate, *Bifidobacterium spp.* primarily promote their intestinal synthesis *via* cross-feeding interactions with other bacteria, such as *Faecalibacterium*, *Eubacterium*, and *Anaerostipes*. These interactions increase utilization of carbon sources, especially prebiotics, and facilitate the production of propionate and butyrate (Gavzy et al., 2023). To quantify the relationship between enriched *Bifidobacterium spp.* in the DDP group and SCFA concentrations, correlation analyses were performed. All SCFAs except valeric acid exhibited strong positive correlations with the enriched *Bifidobacterium spp.* (Fig. 2G). Notably, acetic acid (*r* > 0.95, *p* < 0.01), propionic acid (*r* > 0.90, *p* < 0.05), and butyric acid (*r* > 0.92, *p* < 0.01) exhibited stronger correlations than isobutyric and isovaleric acids. These findings support the hypothesis that DDP enhance SCFA production by modulating the gut microbiota.

**Fig. 3:**
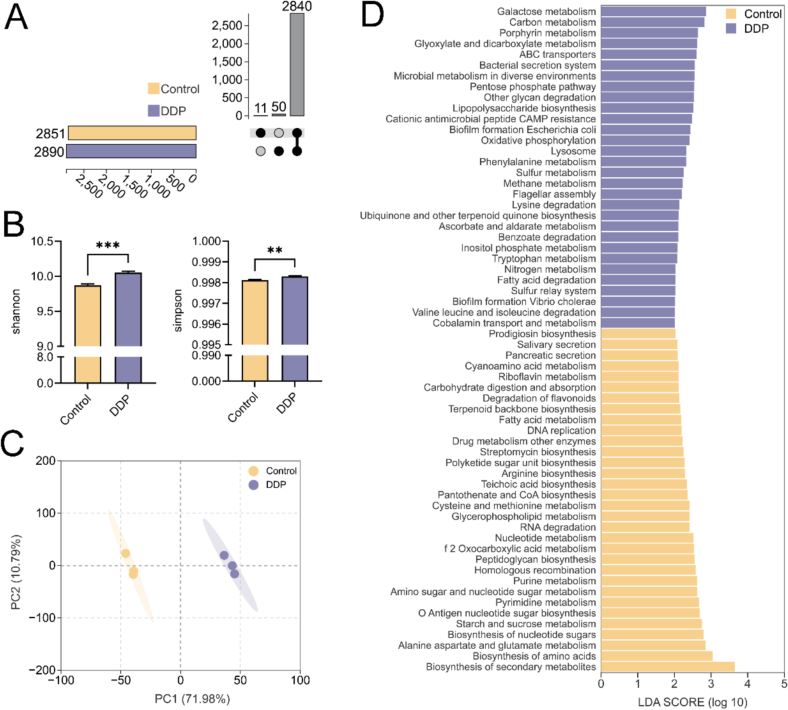
DDP improve the functional characteristics of the gut microbiota. (A) UpSet plot of the functional pathways corresponding to the gut microbiota. (B) Shannon and Simpson indices for the functional pathways of the gut microbiota. (C) PCA of the functional pathway profiles. (D) Differentially abundant functional pathways between groups (LDA score > 2). DDP, polyphenols from *Docynia delavayi* (Franch.) Schneid fruit; SCFAs, short-chain fatty acids; PCA, principal component analysis. **p < 0.01; ***p < 0.001; *n =* 3.

Further investigation into the effect of DDP on gut microbiota involved aligning microbial profiling data with the Kyoto Encyclopedia of Genes and Genomes (KEGG) database to determine functional characteristics. Functional annotation identified 2851 pathways in the Control group and 2890 in the DDP group, with 2840 pathways shared (Fig. 3A). Diversity analysis indicated that DDP significantly increased the Shannon and Simpson diversity indices (Fig. 3B) and elevated the PC1 score in the PCA plot (Fig. 3C), reflecting the trends observed in microbial composition. LEfSe analysis identified differential metabolic pathways between the groups (LDA score > 2; Fig. 3D). In the DDP group, 30 pathways were significantly enriched, including those related to phenolic compound and carbohydrate metabolism. For instance, benzoate degradation and phenylalanine metabolism were both enriched, with the former associated with intermediate metabolites of various phenolic compounds, indicating enhanced metabolism of compounds such as hydroxybenzoic acid and gallic acid (Khadem & Marles, 2010). Meanwhile, phenylalanine metabolism, regulated by phenolic acid metabolites including chlorogenic acid, caffeic acid, and protocatechuic acid, correlated significantly with their levels of synthesis (Yu et al., 2024). The enrichment of these pathways suggests that DDP are efficiently metabolized by the gut microbiota to produce bioactive phenolic acid compounds. Additionally, the ABC transporter pathway was significantly enriched in the DDP group, suggesting enhanced transmembrane transport of phenolic compounds, further supporting efficient DDP metabolism by the gut microbiota (Thomas & Tampé, 2020).

In addition to phenolic compound metabolism, carbohydrate metabolism pathways—such as galactose metabolism, other glycan degradation, carbon metabolism, and the pentose phosphate pathway—were also significantly enriched, aligning with the hypothesis that *Bifidobacterium spp.* enhance carbon source utilization through cross-feeding to promote SCFA synthesis. The glyoxylate and dicarboxylate metabolism pathway was also enriched in the DDP group, suggesting increased conversion of dicarboxylic acids, such as succinate into propionate (Haller et al., 2000). Enrichment of microbial metabolism across diverse environments directly confirmed increased microbial metabolic activity, suggesting an increase in the synthesis of polyphenolic secondary metabolites and SCFAs. Taken together, these alterations in the gut microbiota's functional characteristics suggest that DDP exert a substantial and beneficial regulatory influence on the microbiota.

### 3.5 DDP improved gut microbial metabolism and enriched SCFA-related metabolic pathways

**Fig. 4:**
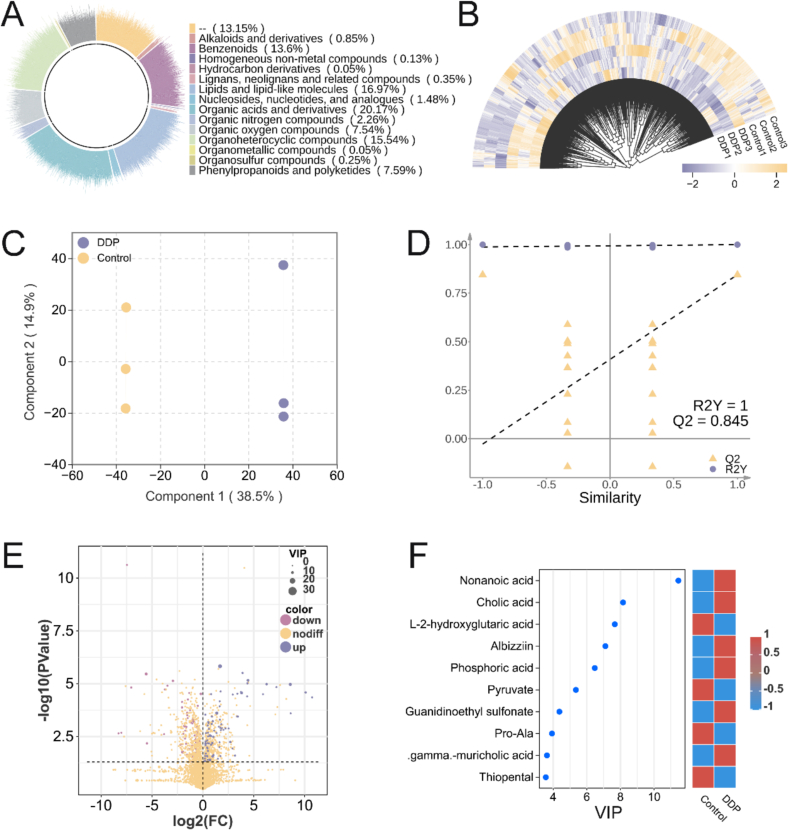
DDP improve the gut microbial metabolic profile. (A) Overview of the major metabolite classes and their relative abundances. (B) Hierarchical clustering heatmap. (C) Orthogonal partial least squares-discriminant analysis (OPLS-DA) score plot. (D) OPLS-DA permutation test results. (E) Volcano plot. (F) Distribution of the top 10 differentially expressed metabolites (based on VIP scores, *p* < 0.0001) across groups. DDP, polyphenols from *Docynia delavayi* (Franch.) Schneid fruit; *n* = 3.

Analysis of the metabolomic profiles revealed that metabolites were classified into 14 major categories, with organic acids and their derivatives comprising the largest proportion (20.17 %). In contrast, organometallic compounds and hydrocarbon derivatives were the least abundant (0.05 %; Fig. 4A). Cluster analysis highlighted clear differences in the metabolite profiles between the Control and DDP groups (Fig. 4B), emphasizing the impact of DDP intervention on gut microbial metabolism. Further multivariate analysis using orthogonal partial least squares-discriminant analysis (OPLS-DA) demonstrated distinct separation between the Control and DDP groups (Fig. 4C). The reliability and accuracy of this model were confirmed by a corresponding permutation test (R^2^ = 1, Q^2^ > 0.5), supporting the robustness of the findings (Fig. 4D). Differentially expressed metabolites were identified using OPLS-DA with Pareto scaling, applying thresholds of variable importance in the projection (VIP) > 1 and *p* < 0.05. In total, 78 metabolites were significantly upregulated, whereas 70 were downregulated in the DDP group compared to the Control group (Fig. 4E). Among the top 10 differentially expressed metabolites (*p* < 0.0001; Fig. 4F), several are recognized for their beneficial physiological effects. Notably, phosphoric acid, which plays a pivotal role in membrane synthesis, signal transduction, and energy metabolism, was markedly increased and positively correlated with the enrichment of *M. jalaludinii* in the DDP group (De Vos et al., 2024).

**Fig. 5:**
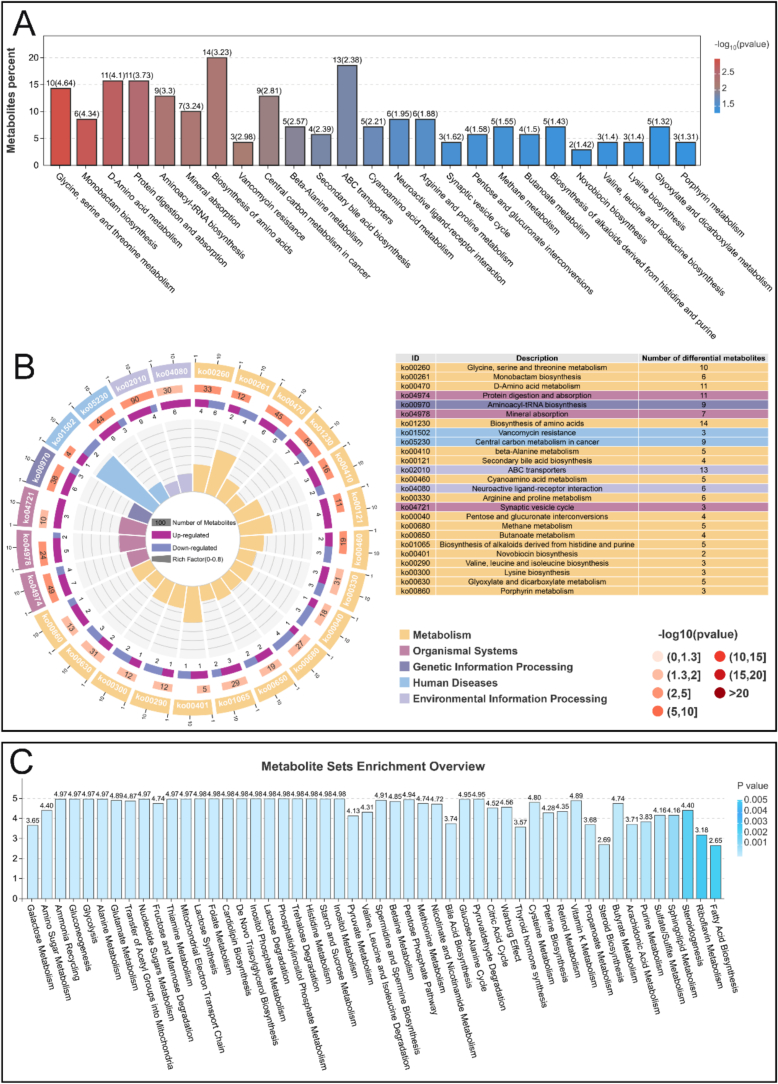
DDP enrich metabolic pathways associated with SCFA synthesis. (A) Kyoto Encyclopedia of Genes and Genomes (KEGG) pathway enrichment analysis; Y-axis represents the percentage of differentially expressed metabolites among the total metabolites detected; bar heights indicate the number of differentially expressed metabolites enriched in each pathway (*p*-values transformed as –log10(p)). (B) Circular plot of KEGG pathway enrichment analysis results. (C) Metabolite set enrichment analysis based on the small molecule pathway database; Y-axis indicates the degree of pathway enrichment. DDP, polyphenols from *Docynia delavayi* (Franch.) Schneid fruit; SCFAs, short-chain fatty acids; *n* = 3.

To elucidate the underlying metabolic pathways affected by DDP, differential metabolites were mapped to the KEGG database. This approach identified 25 pathways that were significantly enriched between the Control and DDP groups (*p* < 0.05; Fig. 5A, B). Notably, butanoate metabolism was significantly enriched in the DDP group, indicating enhanced SCFA synthesis. Additional enrichment was observed in pathways including ABC transporters, glyoxylate and dicarboxylate metabolism, and various protein–amino acid anabolic networks such as glycine, serine, threonine, D-amino acid, and β-alanine metabolism. These pathways are fundamental to cellular growth, metabolic efficiency, and immune regulation, and collectively contribute to SCFA production (Archana et al., 2024). These results support the conclusion that DDP promote SCFA production, likely by modulating gut microbiota function. In addition, the mineral absorption pathway was significantly enriched in the DDP group, suggesting improved intestinal mineral transport. This enhancement may be attributed to the increased abundance of *M. jalaludinii*, which facilitates mineral release through phytate metabolism and contributes to essential physiological processes, including bone and nerve functions, hormonal regulation, and enzymatic activity (De Vos et al., 2024).

Metabolite set enrichment analysis (MSEA) provided further insight into systemic metabolic differences between the Control and DDP groups (Fig. 5C), identifying 50 significantly enriched pathways (*p* < 0.01). Among these, propanoate and butyrate metabolism were notably enriched, further reinforcing the gut microbiota's upregulation of SCFA production. Enrichment in nitrogen and amino acid metabolic networks, including alanine and glutamate metabolism, was also observed and corroborated by KEGG pathway analysis. Additionally, several carbohydrate metabolic pathways, such as galactose metabolism, glycolysis, and starch and sucrose metabolism, were significantly enriched in the DDP group. These carbohydrate metabolic routes are upstream of SCFA biosynthesis (Zhang et al., 2023) and reflect DDP's role in promoting microbial carbohydrate transport and utilization. Lipid metabolism-related pathways, including cardiolipin biosynthesis and sphingolipid metabolism, were also enriched and are closely associated with mitochondrial function, energy homeostasis, and the immune response (Yan & Horng, 2020)—key physiological processes regulated by SCFAs. Pathways involving small bioactive compounds, notably bile acid biosynthesis and retinol metabolism, were also significantly enriched, highlighting DDP's multifaceted regulatory role of DDP in gut microbial metabolism.

DDP significantly enhanced the synthesis of acetic, propionic, and butyric acids, while exerting a comparatively limited effect on valeric acid synthesis. This selective influence is likely due to DDP's targeted remodeling of gut microbial structure and metabolic pathways. SCFA synthesis is dependent on the composition of the gut microbiota and the presence of specific metabolic pathways. Dietary factors can serve as substrates or regulatory signals that selectively stimulate the growth and metabolic activity of SCFA-producing species (Mukhopadhya & Louis, 2025). Acetic, propionic, and butyric acids are abundant in the colon and are synthesized by species such as *Bacteroides* and *Bifidobacterium spp.*, with carbohydrate metabolism playing a central role (Mukhopadhya & Louis, 2025). In contrast, valeric acid is produced at lower levels through distinct pathways, primarily mediated by species such as *Lachnospira* and *Oscillibacter valericigenes*, and is potentially influenced by branched-chain amino acid metabolism (Archana et al., 2024; Iino et al., 2007).

Within this study, DDP supplementation led to a significant increase in the relative abundances of *B. adolescentis*, *B. pseudocatenulatum*, *B. longum*, and *B. bifidum* (Fig. 2F). These species are direct acetic acid producers and enhance carbon source utilization, particularly of prebiotics, through cross-feeding mechanisms, thereby promoting the synthesis of propionic and butyric acids while exerting limited influence on valeric acid production (Vieira et al., 2016). Thus, this *Bifidobacterium*-dominant microbiota structure fostered by DDP supports increased acetic, propionic, and butyric acids synthesis. Furthermore, metabolomic analysis revealed that DDP significantly enriched carbohydrate metabolism pathways, including glycolysis, starch and sucrose metabolism, and galactose metabolism, further supporting the predominance of acetic, propionic, and butyric acids synthesis (Mukhopadhya & Louis, 2025). These findings are consistent with previous research (Zhong et al., 2023).

### 3.6 DDP promoted SCFA production by enriching the chlorogenic acid–caffeic acid–protocatechuic acid metabolic pathway

The biological effects of polyphenols are strongly influenced by their bioavailability, which is largely determined by microbial metabolism in the gastrointestinal tract (Di Lorenzo et al., 2021). Phenolic acids, formed as primary microbial metabolites of polyphenols, possess greater bioavailability than their parent compounds (Ward et al., 2004). The microbial transformation process not only enhances the bioavailability but also increases the physiological activities of polyphenols, such as their antioxidant capacity. As a result, phenolic acid metabolites are widely regarded as major contributors to the biological activities of various polyphenol constituents (Ward et al., 2004).

**Fig. 6:**
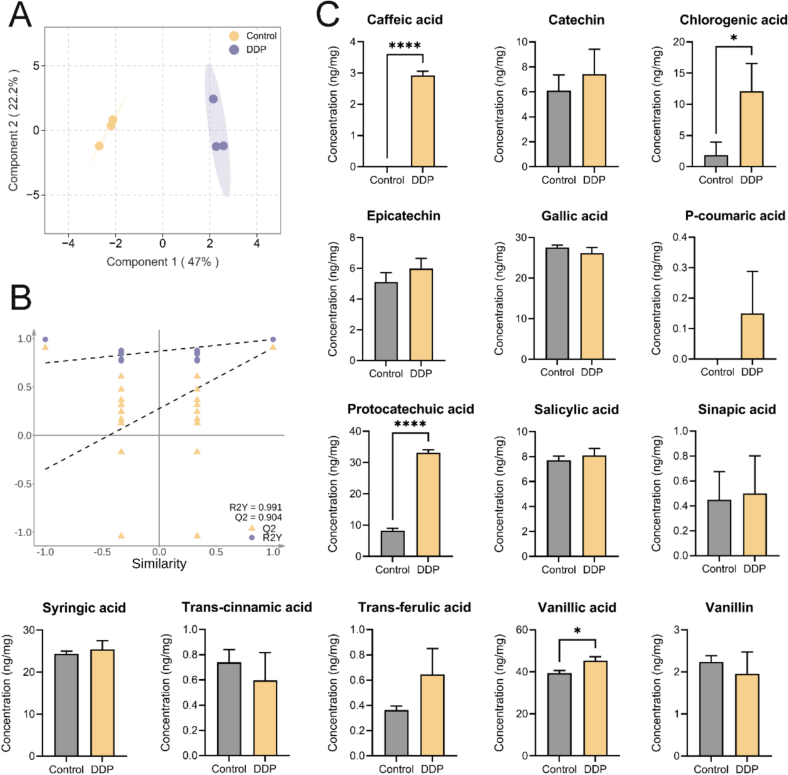
Targeted metabolomics analysis of phenolic acid metabolites. (A) Orthogonal partial least squares-discriminant analysis (OPLS-DA) of the phenolic acid metabolites. (B) OPLS-DA permutation test results. (C) Quantitative results of the phenolic acid metabolites. *p < 0.05; ****p < 0.0001; *n* = 3.

In the present study, targeted metabolomics was employed to quantify representative phenolic acid metabolites in post-fermentation samples from the Control and DDP groups, identifying 14 phenolic acids (Fig. 6). OPLS-DA indicated a clear separation between the two groups (Fig. 6A), and the robustness of the model was confirmed by permutation testing (R^2^ = 0.991, Q^2^ > 0.5; Fig. 6B). Significant increases were observed in chlorogenic acid (*p* < 0.01), caffeic acid (*p* < 0.0001), protocatechuic acid (*p* < 0.0001), and vanillic acid (*p* < 0.05) in the DDP group compared to the Control (Fig. 6C).

**Fig. 7:**
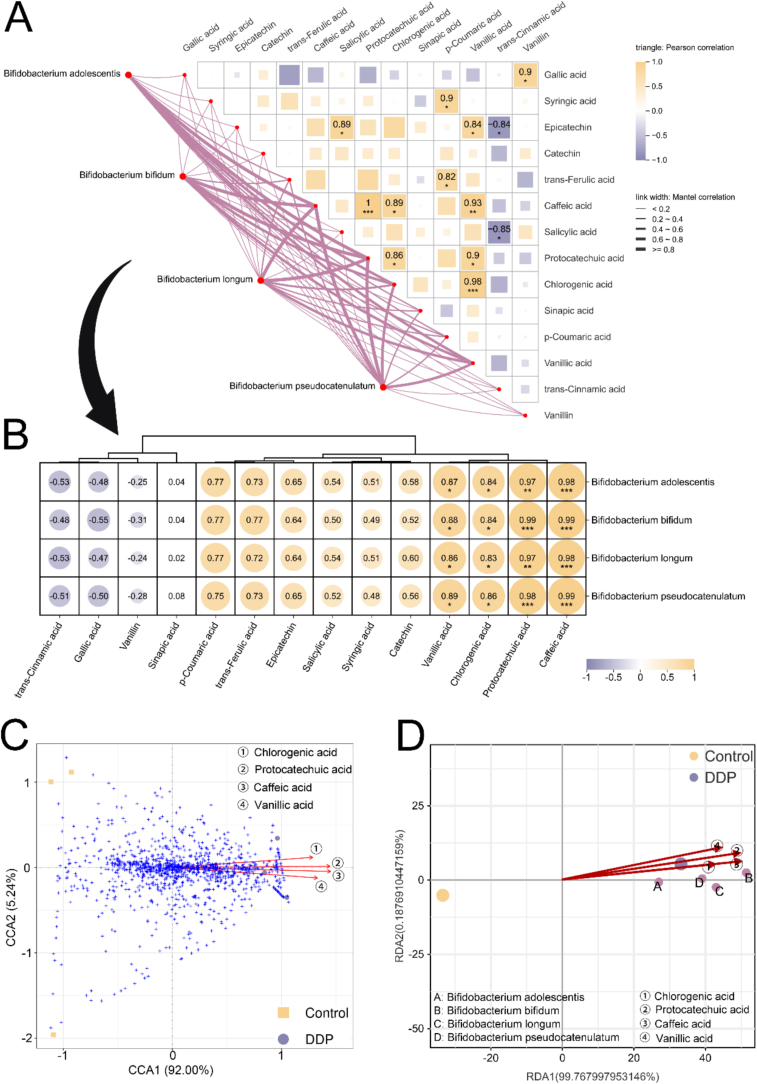
DDP improve gut microbiota composition and stimulates SCFA synthesis by enriching the chlorogenic acid–caffeic acid–protocatechuic acid metabolic pathway. (A) Mantel test heatmap of SCFAs and enriched *Bifidobacterium* species in the DDP group. (B) Correlation analysis of SCFAs and enriched *Bifidobacterium* species in the DDP group. (C) Canonical correspondence analysis of phenolic acid metabolites (caffeic acid, chlorogenic acid, protocatechuic acid, and vanillic acid) and the gut microbiota. (D) Redundancy analysis of SCFAs and enriched *Bifidobacterium* species in the DDP group. DDP, polyphenols from *Docynia delavayi* (Franch.) Schneid fruit; SCFAs, short-chain fatty acids; DSS, dextran sodium sulfate; *n =* 3.

**Fig. 8:**
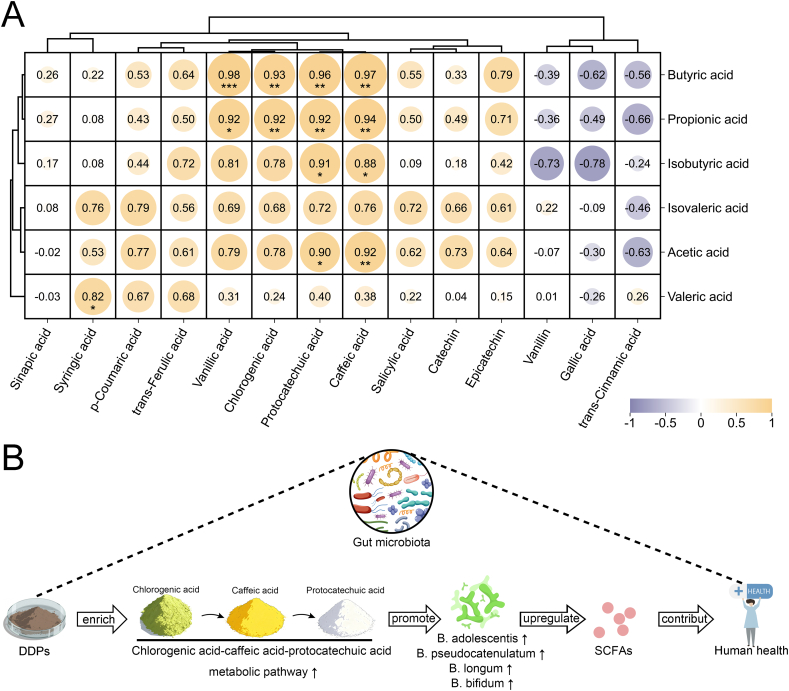
DDP regulate gut microbiota and short-chain fatty acid synthesis *via* the chlorogenic acid–caffeic acid–protocatechuic acid metabolic pathway. (A) Correlation analysis of phenolic acid metabolites (caffeic acid, chlorogenic acid, protocatechuic acid, and vanillic acid) and enriched *Bifidobacterium spp.* in the DDP group; (B) DDP promote SCFA production *via* the chlorogenic acid–caffeic acid–protocatechuic acid metabolic pathway. DDP, polyphenols from *Docynia delavayi* (Franch.) Schneid fruit; *n =* 3.

Building on previous analyses, the observed enrichment of *B. adolescentis*, *B. pseudocatenulatum, B. longum,* and *B. bifidum* in the DDP group was identified as critical for enhancing SCFA synthesis. To further explore the mechanisms underlying this effect, multi-omics analyses quantified the contributions of DDP-derived phenolic acid metabolites to gut microbiota modulation (Fig. 7). Mantel analysis (Fig. 7A) identified 11 strong positive correlations (*r* > 0.80, *p* < 0.05) and 2 strong negative correlations (*r* < −0.80, *p* < 0.05) among the 14 phenolic acids. Notably, four metabolite pairs exhibited strong positive correlations (*r* > 0.90), with no extreme negative correlations detected. Caffeic acid, protocatechuic acid, chlorogenic acid, and vanillic acid were each strongly and positively correlated with *Bifidobacterium spp.* (*r* > 0.80), with caffeic acid and protocatechuic acid demonstrating particularly strong correlations (*r* > 0.90), suggesting a predominant influence (Fig. 7B). To validate these findings, CCA and RDA were performed (Fig. 7C and D). The directional vectors of the four acids traversed dense microbial clusters aligned with the CCA1 axis (92 %), underscoring their significant impact on species composition. Among these compounds, caffeic acid and protocatechuic acid displayed longer vectors than chlorogenic acid and vanillic acid, indicating a stronger effect on the gut microbiota. The spatial proximity—measured as the straight-line distance from arrow segments to the species—further confirmed that chlorogenic acid and caffeic acid had the most direct association with enriched *Bifidobacterium* species. Significant positive correlations were detected between caffeic acid, protocatechuic acid, and multiple SCFAs (acetic, propionic, butyric, and isobutyric acids), while chlorogenic acid and vanillic acid were significantly associated with propionic and butyric acids (Fig. 8A). Collectively, these results support the hypothesis that these phenolic acid metabolites actively promote SCFA synthesis by regulating the gut microbiota, consistent with previously described functional characteristics.

Chlorogenic acid is among the most abundant and bioactive dietary polyphenols, with its key metabolites including caffeic acid, protocatechuic acid, and vanillic acid. Within the gut microbiota, chlorogenic acid undergoes sequential transformations: ester bond hydrolysis produces caffeic acid, subsequent hydrogenation and decarboxylation reactions convert caffeic acid to protocatechuic acid, and methylation of the 3′-hydroxyl group yields vanillic acid (Clifford et al., 2020). Evidence suggests that these rapid structural modifications profoundly alter the bioactivity of the parent compounds (Ward et al., 2004). We previously conducted a detailed characterization of the components of DDP, revealing abundant chlorogenic acid (313.75 ng/mg) but comparatively low levels of caffeic acid (0.35 ng/mg), protocatechuic acid (2.43 ng/mg), and vanillic acid (0.39 ng/mg) (Zhang et al., 2025). However, following *in vitro* digestion and fermentation, the proportions of caffeic, protocatechuic, and vanillic acids increased significantly. Multi-omics analyses further indicated that, compared with chlorogenic acid and vanillic acid, caffeic acid and protocatechuic acid played more prominent roles in modulating the gut microbiota and enhancing SCFA synthesis. This suggests that chlorogenic acid in DDP likely acts primarily through its conversion to caffeic acid and protocatechuic acid. Notably, these metabolites are more readily absorbed than chlorogenic acid, further supporting this mechanism (Heleno et al., 2015).

Both caffeic acid and protocatechuic acid are small molecules with diverse bioactive properties, including antibacterial, antioxidant, antiaging, anticancer, and analgesic effects (Pavlíková, 2023; Song et al., 2020). While direct studies on their roles in gut microbiota regulation are limited, previous research has shown that a corn starch–caffeic acid complex enhances microbial diversity, promotes *Bifidobacterium* proliferation, and increases SCFA production (Zheng et al., 2025), findings that align with those of the current study. Although no studies have directly demonstrated the dietary role of protocatechuic acid in stimulating *Bifidobacterium* for SCFA synthesis, existing evidence shows that it can restore fecal SCFA levels in mice on a high-cholesterol diet (Zhao et al., 2021).

In summary, these findings suggest that DDP consumption enriches the chlorogenic acid–caffeic acid–protocatechuic acid pathway, promotes *Bifidobacterium* growth, enhances SCFA synthesis, and ultimately contributes to immune modulation and anti-inflammatory effects (Fig. 8B).

## 4 Conclusions

This study systematically investigated the effects of DDP supplementation on the gut microbiota and its metabolic functions by utilizing an *in vitro* digestion–colonic fermentation model alongside comprehensive multi-omics analyses. The research specifically focused on elucidating the potential mechanisms through which DDP promote SCFA synthesis. The findings clearly demonstrate that DDP supplementation significantly increases the production of key SCFAs, including acetic, propionic, butyric, and isobutyric acids, within a human fecal microbiota fermentation model. Metagenomic and untargeted metabolomic analyses further validated these findings, revealing that DDP not only optimize the structural composition and functional potential of the gut microbiota but also enhance microbial metabolic activity. Multi-omics data elucidated the underlying mechanism responsible for the observed SCFA upregulation. The conversion of chlorogenic acid into its active metabolites—caffeic acid and protocatechuic acid—was shown to stimulate the growth *Bifidobacterium spp.* associated with SCFA production, thereby supporting improved intestinal health. In summary, this study establishes a theoretical foundation for the probiotic effects of DDP, emphasizing the central mechanism involving dietary polyphenol transformation into phenolic acid metabolites, subsequent modulation of the gut microbiota, and enhanced SCFA synthesis. These insights offer new scientific perspectives for applying natural products to promote intestinal health.

## CRediT authorship contribution statement

**Tingting Zhang:** Writing – original draft, Visualization, Validation, Methodology, Investigation, Formal analysis. **Mingxia Xing:** Investigation. **Hui Zhang:** Methodology. **Xin Song:** Methodology. **Zibo Song:** Funding acquisition. **Chunmei Yuan:** Resources. **Jun Zhang:** Project administration. **Zhou Zhang:** Writing – review & editing, Resources, Conceptualization. **Fan Xie:** Writing – review & editing, Resources, Funding acquisition, Conceptualization. **Lianzhong Ai:** Supervision.

## Declaration of competing interest

The authors declare that they have no known competing financial interests or personal relationships that could have appeared to influence the work reported in this paper.

## Data Availability

Data will be made available on request.

## Appendix A Supplementary data

Supplementary material Image 1
