# Supplementary material for: Docynia delavayi polyphenols enhance short-chain fatty acid synthesis via the chlorogenic acid–caffeic acid–protocatechuic acid pathway: insights from in vitro digestion–fermentation
Source: Food Chem X. 2025 Dec 16;33:103416. doi: 10.1016/j.fochx.2025.103416 (PMC12807836; doi:10.1016/j.fochx.2025.103416)
Supplement: Supplementary file 1 — Supplementary material [file mmc1.docx]

***Docynia delavayi* Polyphenols Enhance Short-Chain Fatty Acid Synthesis via the Chlorogenic Acid–Caffeic Acid–Protocatechuic Acid Pathway: Insights from *In Vitro* Digestion–Fermentation**

Tingting Zhang^a^, Mingxia Xing^a^, Hui Zhang^a^, Xin Song^a^, Zibo Song^d^, Chunmei Yuan^d^, Jun Zhang^c^, Lianzhong Ai^a, b^, Zhou Zhang^c^*, Fan Xie^a, b^*

^a^Shanghai Engineering Research Center of Food Microbiology, School of Health Science and Engineering, University of Shanghai for Science and Technology, Shanghai, China

^b^Department of Food Science & Technology, School of Agriculture and Biology, Shanghai Jiao Tong University, 800 Dongchuan Road, Shanghai, China

^c^Shidong Hospital, Yangpu District, Shanghai, China

^d^Yunnan Provincial Key Laboratory of Applied Technology for Special Forest Fruits, Yunnan Maoduoli Group Food Co., Ltd., Yuxi, China

* Corresponding authors:

Zhou Zhang: E-mail: 34806375@qq.com

Fan Xie: xiefan246141@163.com.

**Supplementary Material**

Table S1. The modified yeast casitone fatty acids (YCFA) broth.

| Components | Content (g) |
| --- | --- |
| Casitone | 10.00 g |
| Yeast extract | 2.50 g |
| Glucose·H_2_O | 11.00 g |
| MgSO_4_·7H_2_O | 45.00 mg |
| CaCL_2_·2H_2_O | 90.00 mg |
| K_2_HPO_4_ | 0.45 g |
| KH_2_PO_4_ | 0.45 g |
| NaCl | 0.90 g |
| Resazurin | 1.00 mg |

Perform the following steps:

(1) Add 0.5 mL of volatile fatty acid solution and heat to a boil for 10 min.

(2) Rapidly cool to 25°C and purge with carbon dioxide.

(3) Add 1.00 g of L-cysteine hydrochloride, 4.00 g of sodium bicarbonate, and 10.00 mg of hemin.

(4) Adjust the pH to 6.70.

(5) Under nitrogen, aliquot the medium into Hungate tubes (10 mL/tube).

(6) Sterilize at 121°C for 15 min.

(7) After cooling, add filtered sterilized vitamin solution (100 μL/tube).

Table S2. Phenolic acids detected in targeted metabolomics.

| Name | Retention time | *m/z* | Molecular Weight (Da) | CAS id | Formula |
| --- | --- | --- | --- | --- | --- |
| Caffeic acid | 4.58 | 179.1 | 180.16 | 331-39-5 | C_9_H_8_O_4_ |
| Catechin | 3.79 | 289.1 | 290.27 | 154-23-4 | C_15_H_14_O_6_ |
| Chlorogenic acid | 3.81 | 353.1 | 354.31 | 327-97-9 | C_16_H_18_O_9_ |
| Epicatechin | 5.38 | 289.1 | 290.27 | 490-46-0 | C_15_H_14_O_6_ |
| Gallic acid | 1.27 | 168.9 | 170.12 | 149-91-7 | C_7_H_6_O_5_ |
| p-Coumaric acid | 6.77 | 163 | 164.16 | 501-98-4 | C_9_H_8_O_3_ |
| Protocatechuic acid | 2.29 | 153.1 | 154.12 | 99-50-3 | C_7_H_6_O_4_ |
| Salicylic acid | 7.58 | 137.1 | 138.12 | 69-72-7 | C_7_H_6_O_3_ |
| Sinapic acid | 7.2 | 223.1 | 224.21 | 530-59-6 | C_11_H_12_O_5_ |
| Syringic acid | 4.88 | 197 | 198.17 | 530-57-4 | C_9_H_10_O_5_ |
| trans-Cinnamic acid | 7.83 | 149 | 148.16 | 140-10-3 | C_9_H_8_O_2_ |
| trans-Ferulic acid | 7.13 | 193 | 194.18 | 537-98-4 | C_10_H_10_O_4_ |
| Vanillic acid | 4.5 | 149 | 168.15 | 121-34-6 | C_8_H_8_O_4_ |
| Vanillin | 6.27 | 153 | 152.15 | 121-33-5 | C_8_H_8_O_3_ |
